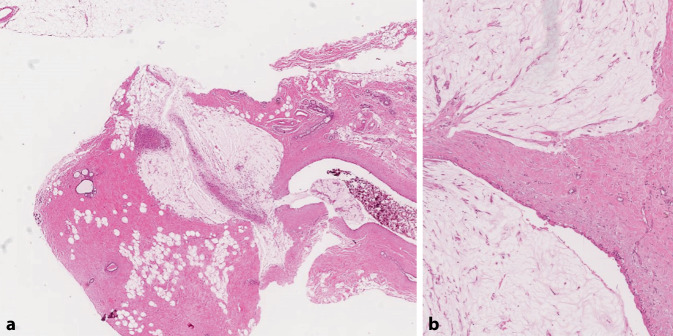# B3-Läsionen der Mamma: Histologische, klinische und epidemiologische Aspekte: Update

**DOI:** 10.1007/s00292-022-01180-3

**Published:** 2023-01-12

**Authors:** Zsuzsanna Varga, Peter Sinn, Annette Lebeau

**Affiliations:** 1 Institut für Pathologie und Molekularpathologie, Universitätsspital Zürich, Schmelzbergstr. 12, 8091 Zürich, Schweiz; 2 Pathologisches Institut, Universitätsklinikum Heidelberg, Heidelberg, Deutschland; 3 Institut für Pathologie, Universitätsklinikum Hamburg-Eppendorf, Hamburg, Deutschland; 4 Gemeinschaftspraxis für Pathologie, Lübeck, Deutschland

**Keywords:** Mamma, Vorläuferläsionen, Atypie, Unterschätzungsrate, Immunhistochemie, Breast, Premalignant lesions, Atypia, Underestimation rate, Immunohistochemistry

## Abstract

B3-Läsionen der Mamma stellen eine heterogene Gruppe von Läsionen mit unsicherem Malignitätspotenzial dar, welche histologisch klar definierte Diagnosen beinhalten und welche in diagnostischem und therapeutischem Kontext unterschiedlich gewichtet und behandelt werden. Vor allem die Entscheidung über die Schritte nach der Diagnose einer B3-Läsion an Stanzbiopsie oder Vakuumbiopsie können eine Herausforderung darstellen. B3-Läsionen werden oft wegen bildgebenden Kalzifikationen oder Herdbefunden entdeckt und biopsiert. Histologisch können die Veränderungen einer Reihe von häufigen Diagnosen (wie die atypische duktale Hyperplasie, die klassische lobuläre Neoplasie, flache Epithelatypien, intraduktale Papillome, radiäre Narben oder fibroepitheliale Tumoren vom Typ eines Phylloidestumors) oder seltenen Diagnosen (wie Mukozelen-ähnliche Läsion, atypische apokrine Läsionen und seltene stromale Proliferate) zugeordnet werden. Immunhistochemie ist hilfreich in der Differenzierung und einer korrekten nomenklatorischen Zuordnung dieser Läsionen (vor allem Östrogenrezeptoren, basale Zytokeratine, Myoepithelmarker oder stromale Markerpanel). Im klinischen Kontext bleibt die Korrelation der bildgebenden und histologischen Befunde der wichtigste Faktor in der Entscheidung der nächsten diagnostischen/therapeutischen Schritte.

## Abstract (en)

B3 lesions of the breast are a heterogeneous group of lesions with uncertain malignant potential encompassing a broad spectrum of histologically distinct alterations that often pose challenging decisions if diagnosed on the preoperative core or vacuum biopsies. B3 lesions are mostly detected due to mammographic calcifications or mass lesions and, in most cases, encompass a spectrum of atypical lesions such as atypical ductal hyperplasia, classic lobular neoplasia, flat epithelial atypia, papillomas, fibroepithelial tumors, and rarely other lesions such as mucocele-like lesions, atypical apocrine lesions, and rare stromal proliferations. The use of immunohistochemical stains (estrogen receptors, basal cytokeratin, myoepithelial markers, and stromal marker panel) is useful in the differentiation of these lesions and allowing proper classification. Regarding clinical management of B3 lesions, the radiological–pathological correlation of the given entity plays the most important key element for the proper next diagnostic and therapeutic step.

Innerhalb der B‑Klassifikation, die 5 Kategorien zur Einordnung der histologischen Veränderungen in diagnostischen Nadelbiopsien umfasst, stellen die B3-Läsionen der Mamma eine heterogene Gruppe von histologisch definierten Erkrankungen dar [1, 16, 21, 40, 47]. Entweder handelt es sich um Läsionen mit Risiko eines assoziierten duktalen Carcinoma in situ (DCIS) oder invasiven Karzinoms oder um Entitäten mit Risiko eines unvollständigen Samplings [1, 40, 46, 55].

Diese Entitäten zeigen zum Teil überlappende Eigenschaften sowohl morphologisch als auch radiologisch, was die nachfolgenden nächsten diagnostischen Schritte erschweren kann [1, 16, 40, 46, 47, 55]. Den B3-Läsionen wird ein unsicheres biologisches Potenzial zugeordnet, weswegen eine interdisziplinäre Besprechung der jeweiligen Entitäten und eine regelmässige Anpassung der klinischen Empfehlungen zum therapeutischen Vorgehen in Abhängigkeit der aktuellen Datenlage erforderlich sind [1, 40]. Eine besondere Herausforderung ist es, eine unnötige Übertherapie zu vermieden [1, 40]. Dementsprechend haben sich die Handlungsempfehlungen während der letzten Jahre zum Teil zugunsten einer Deeskalation verändert [1, 40].

In dieser Zusammenstellung werden die häufigsten B3-Läsionen aus der histopathologischen Sicht angegangen und neben den morphologischen Merkmalen auch die aktuelle Datenlage der Unterschätzungsrate erläutert: atypische duktale Hyperplasie (ADH), klassische lobuläre Neoplasie (LN), flache Epithelatypien (FEA), radiäre Narbe/komplexe sklerosierende Läsion (RS), intraduktale Papillome ohne Atypien (PL) und Phylloidestumoren (benigne und Borderline-Variante PT). Seltenere B3-Läsionen wie mukozeleähnliche Läsionen, atypische apokrine Adenose und blande stromale myofibroblastäre Proliferationen werden nur kurz erläutert.

Detaillierte Entitäten sind in Tab. 1 aufgelistet.

**Table 1:** 

Art der Läsionen		Klinische Konsequenzen	
*1. Läsionen mit Risiko eines assoziierten DCIS oder invasiven Karzinoms*			
a)	Atypische duktale Hyperplasie (ADH) bzw. atypische Epithelproliferation vom duktalen Typ (in Abhängigkeit von der Ausdehnung ggf. B4)	a)	Exzision
b)	Flache epitheliale Atypie (FEA)	b)	Exzision nur bei ausgedehnten Verkalkungen und bei Diskordanz zur Bildgebung
c)	Klassische lobuläre Neoplasie (LN; ALH und LCIS)	c)	Exzision bei Diskordanz zur Bildgebung
d)	Atypische apokrine Adenose	d)	Offene Exzision
*2. Läsionen mit Risiko eines unvollständigen Samplings*			
a)	Zellreiche fibroepitheliale Läsion oder Phylloidestumor ohne Malignitätsverdacht	a)	Exzision
b)	Intraduktales Papillom ohne/mit Atypien, nicht sicher vollständig entfernt (bei Atypien in Abhängigkeit von der Ausdehnung ggf. B4)	b)	Exzision (evtl. auch mittels Vakuumbiopsie, wenn keine Atypien)
c)	Radiäre Narbe bzw. komplexe sklerosierende Läsion (Ausnahme: wenn radiäre Narbe nicht Ursache der radiologischen Veränderung, dann inzidentelle Läsion/B2)	c)	Exzision (evtl. auch mittels Vakuumbiopsie, wenn keine Atypien)
d)	Hämangiom	d)	Exzision
e)	Atypische vaskuläre Läsion	e)	Exzision
*3. Seltene Veränderungen*			
a)	Adenomyoepitheliom	a–f)	Exzision
b)	Mamillenadenom		
c)	Syringomatöser Tumor		
d)	Mikroglanduläre Adenose		
e)	Mukozelenartige Läsion		
f)	Noduläre Fasziitis		
g)	Fibromatose vom Desmoidtyp		
h)	Unklare Spindelzellläsion		

*ALH* atypische lobuläre Hyperplasie*, DCIS* duktales Carcinoma in situ, *LCIS* lobuläres Carcinoma in situ

## Atypische duktale Hyperplasie

**Figure 1:**
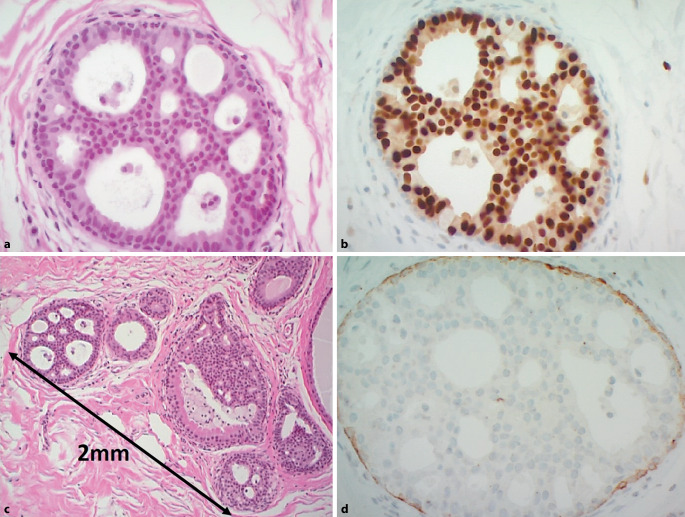

Eine atypische duktale Hyperplasie (ADH; Abb. 1) ist definiert als eine klonale low-grade intraduktale Proliferation, die eine maximale Ausdehnung von 2 mm nicht überschreitet [16, 40, 47, 55]. Häufig, aber nicht immer sind assoziierte Verkalkungen dabei bildgebend zu identifizieren [40, 55]. Ergänzende immunhistochemische Färbungen sind hilfreich für die Diagnosebestätigung. Eine ADH exprimiert stark und diffus Östrogenrezeptoren in 100 % der Zellkerne und zeigt dabei einen Verlust der basalen Zytokeratine (CK5/6 oder CK14) [16, 40, 41, 47]. Die internationale übliche Bezeichnung, die auch in der WHO-Klassifikation geführt wird, ist die „ADH“ wobei in einigen europäischen Ländern auch der Begriff „AIDEP“ („atypical intraductal epithelial proliferation“) gebraucht wird. Als Grund wird angeführt, dass in der Regel an der Nadelbiopsie die endgültige Ausdehnung der atypischen intraduktalen Epithelproliferation nicht festgelegt werden kann und somit keine sichere Grenzziehung zwischen ADH und low-grade duktalem Carcinoma in situ (DCIS) möglich ist [4, 16, 47, 55]. Zahlreiche Studien haben versucht morphologische Kriterien an den präoperativen Biopsien zu identifizieren, die voraussagen können, welche ADH-Fälle im nachfolgenden chirurgischen Präparat höhergradige Läsionen (wie DCIS, invasives Karzinom) oder andere B3-Läsionen wie klassische lobuläre Neoplasie (LN) oder flache epitheliale Atypie (FEA) enthalten [34, 38, 41]. Diese Kriterien (unter anderem die Assoziation zu Verkalkungen, die Zahl der betroffenen Gangstrukturen oder der Nachweis von assoziierten intraläsionalen Nekrosen) können zwar in Einzelfällen prädiktiv sein, eine signifikante Assoziation zwischen diesen Faktoren an der Stanzbiopsie und den Diagnosen im Operationspräparat konnte aber bisher nicht etabliert werden [34, 38, 41]. Es gibt keine radiologischen Merkmale, die helfen, mit ADH assoziierte Verkalkungen von denen anderer Läsionen zu unterscheiden, wie z. B. Kolumnarzellläsionen oder low-grade DCIS [4].

In Zusammenschau der Literaturdaten der letzten Jahre ist das Unterschätzungsrisiko bei einer ADH immer noch hoch und je nach Studie liegt die Upgraderate bei 10–50 % im nachfolgenden Exzisat [3, 11, 15, 17, 29, 35, 37, 41, 52]. Risikofaktoren sind dabei die fehlende Korrelation zwischen bildgebendem Befund und Pathologie, grössere/ausgedehntere bildgebende Befunde oder Herdbefunde und die Art der Biopsie (Diagnose der ADH an Stanz- oder Vakuumbiopsie) [4, 34, 38, 41].

Das kumulative Krebsrisiko eines Mammakarzinoms (ipsilateral und kontralateral) liegt bei ungefähr 30 % nach 25 Jahren [14].

Basierend auf der aktuellen Datenlage ist die offene Befundexzision nach einer bioptischen ADH-Diagnose zu empfehlen, wobei in Einzelfällen auch eine bildgebende Kontrolle gerechtfertigt ist [1, 40, 46, 55].

## Klassische lobuläre Neoplasie

**Figure 2:**
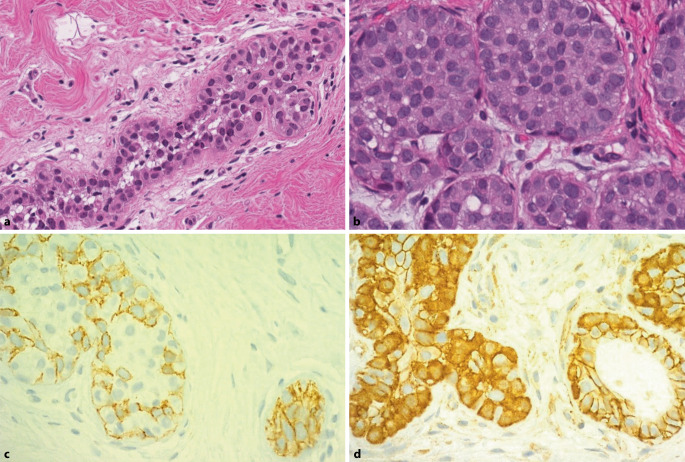

Eine klassische lobuläre Neoplasie (Abb. 2) ist definiert als eine diskohäsive, kleinzellige Epithelproliferation, die von den terminalen duktulolobulären Einheiten (TDLU) ausgeht [18, 25]. Sie wird gemäss der WHO-Klassifikation (2019) unterteilt in lobuläres Carcinoma in situ (LCIS) und in atypische lobuläre Hyperplasie (ALH) [18, 25, 55]. Biologisch gelten sowohl das klassische LCIS als auch die ALH als nichtobligate Vorläuferläsionen des invasiven Mammakarzinoms [18, 25, 55]. Die Unterscheidung zwischen klassischem LCIS und ALH hängt vor allem von deren Ausdehnung (< 50 % >) innerhalb der TDLU-s ab, wobei auf molekularer Ebene LCIS und ALH keine signifikanten Unterschiede aufweisen und mit einer ungenügenden diagnostischen Reproduzierbarkeit einhergehen [31, 55]. Die Differenzierung zwischen ALH und LCIS ist an Nadelbiopsien oft nicht möglich, da die TDLU-s nicht in vollem Umfang erfasst sind. Die Verwendung des Begriffes lobuläre Neoplasie ist daher gerade an Nadelbiopsien weiterhin möglich. Die wichtigste molekulare Alteration ist der Verlust der Zelljunktionsproteine (E-Cadherin, β‑Catenin, Catenin p120), der in bis zu 80 % der LN-Fällen vorkommt und für die Diagnosesicherung immunhistochemisch verwendet werden kann [18, 25, 55]. Abzugrenzen von klassischem LCIS und ALH (beide B3-Läsionen) sind die nicht-klassischen LCIS-Subtypen (floride oder pleomorph), welche analog zum DCIS als B5a klassifiziert werden [1, 40].

Die klassische LN präsentiert sich meist als inzidenteller Befund, eher selten mit assoziierten Verkalkungen einhergehend [23, 28]. In bis zu 5 % der präoperativen Biopsien wird die klassische LN diagnostiziert [20, 23]. Dagegen gehen das floride und pleomorphe LCIS häufig mit Verkalkungen als Folge von Komedonekrosen einher und werden mammographisch entdeckt [55].

Die aktuelle Datenlage zeigt ein variables Unterschätzungsrisiko bei einer klassischen LN-Diagnose. Die Upgraderate am nachfolgendem Operationspräparat variiert in der Literatur in Abhängigkeit von Studie und Nomenklatur zwischen 0 und 60 % [9, 18, 25, 55]. Vor allem bei fehlender radiologisch-pathologischer Korrelation ist mit höheren Upgraderaten zu rechnen, weshalb die radiologisch-pathologische Diskordanz bis dato als wichtigster Risikofaktor für die Unterschätzung angesehen wird [9, 12, 23, 49, 55]. Das relative Risiko, später an Brustkrebs zu erkranken, ist bei Patientinnen mit klassischem LCIS 8‑ bis 10-mal höher als in der Allgemeinbevölkerung. Das absolute Brustkrebsrisiko bei klassischer LN liegt bei 1–2 % pro Jahr mit einer kumulativen Langzeitrate von > 20 % nach 20 Jahren und einem Lebenszeitrisiko von 30–40 % [18, 25, 49, 55]. Zusätzlich sind das jüngere Alter und assoziierte Verkalkungen als weitere Risikofaktoren anzusehen [18, 25, 55]. Basierend auf der aktuellen Datenlage hängen die Therapiemodalitäten von der Art der lobulären Neoplasie und der radiologisch-pathologischen Korrelation ab. Bei isoliertem oder inzidentellem Befund einer LN (klassische Variante) in der Stanz- oder Vakuumbiopsie und Konkordanz mit der Bildgebung kann auf eine weitere bioptische Abklärung verzichtet werden. Bei LN mit erhöhtem Risiko (pleomorphe LN, floride LN oder LN mit Komedotypnekrosen) sollte eine Exzision der Veränderung durchgeführt werden, ebenso bei Diskordanz zum radiologischen Befund [18, 25, 55]. Diese LN mit erhöhtem Risiko gehen mit einer erhöhten Wahrscheinlichkeit einer Mikroinvasion einher [45].

## Flache Epithelatypie

**Figure 3:**
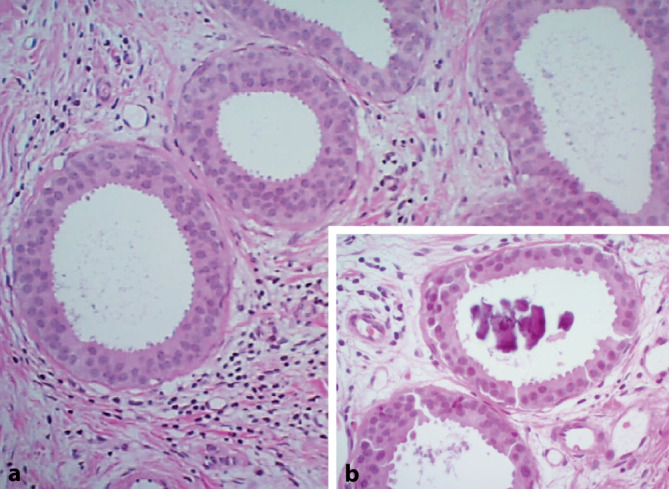

Die flache Epithelatypie (FEA; Abb. 3) gehört der Gruppe der Kolumnarzellläsionen an. Sie wird definiert als monotone low-grade intraduktale atypische Epithelproliferation, welche häufig kalkassoziiert ist und in der Regel nicht mehr als 3–4 Zelllagen hat [16, 47, 55]. Eine FEA ist von nicht-atypischen Kolumnarzellläsionen abzugrenzen, welche als B2 klassifiziert werden [1, 40, 50, 55]. Besonderheit der FEA ist ihre Assoziation zu tubulären Karzinomen und zu anderen B3-Läsionen (wie ADH, klassische LN) oder zu low-grade DCIS, wonach vor allem in den präoperativen Biopsien zu suchen ist [1, 16, 40, 47, 55]. Eine FEA ist eine klonale Läsion mit identischem immunhistochemischem Expressionsprofil (homogen ER-positiv, keine Expression der basalen Zytokeratine) wie eine ADH, aber ohne Ausbildung komplexer Architekturmuster [16, 47, 55]. Radiologisch präsentiert sich die FEA häufig mit assoziierten Verkalkungen. Das relative Risiko für eine Brustkrebsentwicklung ist 1,5fach erhöht. Letztlich hängt das Risiko wie bei den papillären Läsionen aber in erster Linie von potenziell begleitenden anderen B3-Läsionen oder malignen/prämalignen Entitäten ab [55]. Die Upgraderate im nachfolgenden Operationspräparat bei reinen FEA-Fällen liegt deutlich unter 10 % [6, 10, 54]. Basierend auf der aktuellen Datenlage wird bei reiner FEA und bei kompletter radiologisch-pathologischer Korrelation zunehmend ein konservatives Vorgehen gewählt. Auf eine offene PE kann verzichtet werden, wenn suspekte Verkalkungen mittels Vakuumbiopsie bildgebend bereits vollständig oder weitestgehend vollständig entfernt wurden. Eine offene Exzision sollte bei FEA-Fällen mit Diskrepanz zur Bildgebung und bei sehr ausgedehnten Verkalkungen erfolgen [1, 2, 13, 24, 27, 30, 40].

## Radiäre Narbe/komplexe sklerosierende Läsion

**Figure 4:**
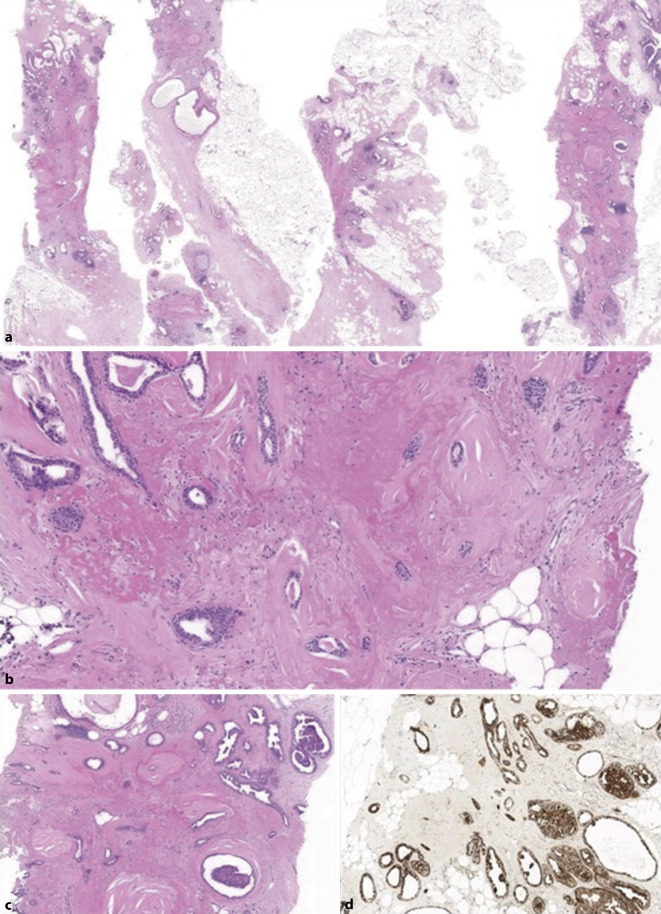

Ein radiäre Narbe („radial scar“, RS) oder in Abhängigkeit von derer Ausdehnung (< 1–1,5 cm >) eine komplexe sklerosierende Läsion (KSL) ist definiert als eine zentrale sternförmige Fibroelastose begleitet von peripheren Zysten, peripheren proliferativen Läsionen und Verkalkungen (Abb. 4; [42, 55]). In einer kleinen Prozentzahl der RS/KSL können atypische Proliferate oder maligne bzw. prämaligne Läsionen (wie DCIS, invasives Karzinom) vorkommen [55]. Immunhistochemie ist äusserst hilfreich in der Differenzierung der RS/KSL von invasiven Karzinomen und der Einordnung der begleitenden intraduktalen Epithelproliferationen. Hier kommt vor allem die immunhistochemische Darstellung der Myoepithelien mit diversen Markern (u. a. p63, basale Zytokeratine) und die Abklärung der intraduktalen/intraazinären Proliferate mit basalen Zytokeratinen und dem Östrogenrezeptor zur Anwendung [42, 55]. Auch bildgebend kann eine RS/KSL ein invasives Mammakarzinom imitieren [39, 40, 48].

Gemäss aktueller Datenlage ist bei einer RS/KSL ohne Atypien im Allgemeinen mit niedrigem Unterschätzungsrisiko zu rechnen, welches sich bei zwischen 0 bis 10 % bewegt [36, 39, 40, 48]. Hingegen gehen RS/KSL mit assoziierten atypischen Läsionen (wie ADH) mit einem höheren Unterschätzungsrisiko (5–30 %) einher [5, 6].

Auch bei dieser Läsion ist die Diskordanz zwischen Pathologie und Radiologie der wichtigste Risikofaktor für Unterschätzung [39, 40, 48]. Die Behandlung von mammographisch festgestellten RS/KSL ist nach wie vor umstritten, sofern keine intraduktale Atypien vorliegen, wobei bei RS/KSL ohne Atypien eine vakuumbioptische Befundentfernung ausreichend zu sein scheint [1, 36, 40]. Läsionen, bei denen nadelbioptisch Atypien festgestellt wurden, sollten aufgrund des Karzinomrisikos entfernt werden [55].

## Papilläre Läsionen

**Figure 5:**
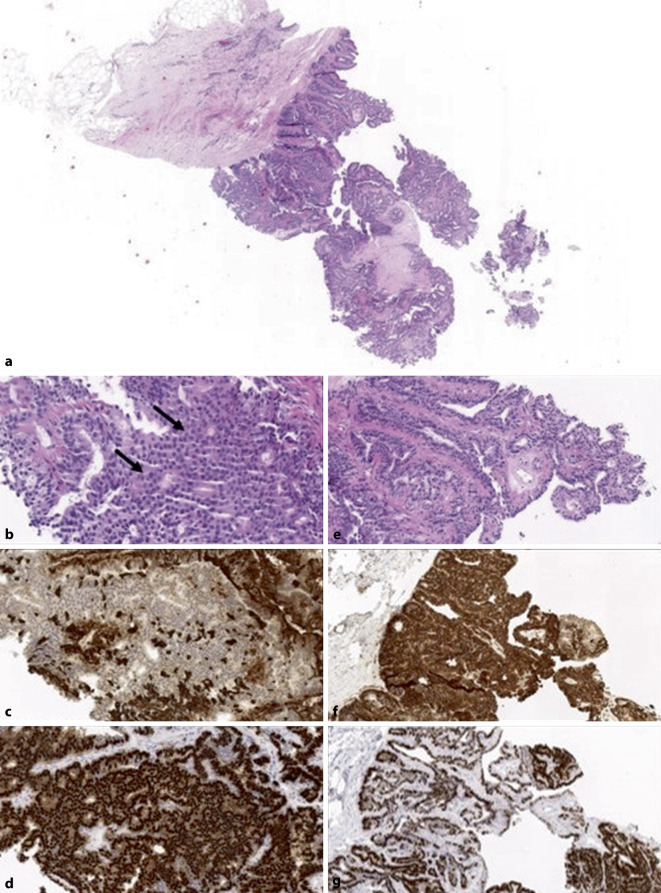

Papilläre Läsionen (PL) stellen ein breites Spektrum von histologischen Läsionen dar, welche das intraduktale Papillom, das Papillom mit Atypien (wie ADH, LN; Abb. 5), das Papillom mit DCIS, das gekapselte papilläre Karzinom und das papilläre DCIS sowie das solid-papilläre Karzinom beinhalten [16, 47, 51, 55]. Die B‑Klassifikation variiert entsprechend. In Abhängigkeit von der Ausdehnung und von den histologischen Eigenschaften werden die nachgewiesene Veränderung als B2 (Mikropapillome, in einem Zylinder in vollem Umfang erfasst), B3 (Papillome mit/ohne Atypien) und B5a (Papillome mit DCIS, papilläres DCIS und enkapsuliertes papilläres Karzinom) eingeordnet [1]. Bei solid-papillären Karzinomen, die häufig neuroendokrine Marker exprimieren (Chromogranin A, Synaptophysin) kann eventuell eine B5c-Kategorie angezeigt sein, wenn eine Differenzierung zwischen einem In-situ-Karzinom und einem invasiven Karzinom an der Nadelbiopsie nicht möglich ist [55]. Im Gegensatz zur ADH ausserhalb einer papillären Läsion, für die als Obergrenze 2 mm definiert wurde, beträgt die maximale Ausdehnung einer ADH innerhalb eines Papilloms gemäss aktueller WHO-Klassifikation 3 mm [55]. Intraduktale Papillome sind klar definierte papilläre Proliferate, welche sich um filigrane fibrovaskuläre Stielelemente ordnen und eine baumähnliche verzweigte Struktur bilden [51, 55]. Innerhalb der Papillome sind definitionsgemäss gleich viel epitheliale wie myoepitheliale Zellen vorhanden, welche immunhistochemisch (myoepitheliale und luminale epitheliale Marker) überprüft werden können [1, 51]. Eine begleitende gewöhnliche intraduktale Proliferation (meist UDH, seltener ADH) lässt sich immunhistochemisch durch basale Zytokeratine, myoepitheliale Marker und Östrogenrezeptoren in der überwiegenden Zahl der Fälle zuverlässig abgrenzen [1, 16, 40, 46, 47, 51, 55]. Eine mosaikartige CK5/6- oder CK14-Expression und eine heterogene Östrogenrezeptorexpression untermauern eine UDH-Diagnose innerhalb der Papillome und grenzen sie von einer ADH ab [16, 47, 51, 55].

Die aktuelle Datenlage zeigt, dass reine Papillome (ohne Atypien) eine niedrige Assoziation mit höhergradigen Läsionen haben. Das Unterschätzungsrisiko liegt unter 10 % [1, 40, 51, 55]. Hingegen weisen Papillome mit Atypien (meist ADH) eine deutlich höhere Unterschätzungsrate (bis 30–40 %) auf [40, 55]. Literaturdaten deuten darauf hin, dass periphere Papillome, die häufig multiple auftreten, im Vergleich zu zentralen, früh symptomatischen Papillomen mit einem höheren Brustkrebsrisiko einhergehen [19, 26, 32, 33, 40, 44]. Bei Papillomen ohne Atypien ist das relative Risiko für Brustkrebs nur gering erhöht (2fach) im Gegensatz zu Papillomen mit Atypien bei denen das Risiko deutlich höher ist (5- bis 7fach) [19, 22, 26, 32, 33, 44]. Die Therapiemodalitäten bei intraduktalen Papillomen richten sich in erster Linie nach dem Fehlen oder Vorhandensein begleitender Atypien. Bei reinen Papillomen ohne Atypie nach vollständiger Entfernung mittels Vakuumbiopsie ist zunehmend die Tendenz einer konservativen Therapie mit bildgebender Kontrolle anzugehen [1, 40, 55].

## Phylloidestumor (benigne und bordeline Variante)

**Figure 6:**
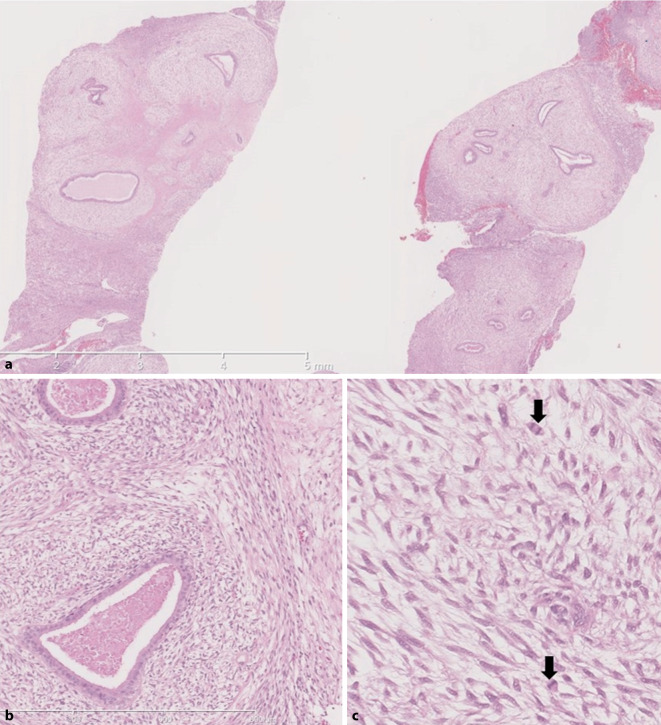

Histologisch wird ein Phylloidestumor (PT, Abb. 6) aus blattartigen Strukturen aufgebaut, die eine epitheliale und stromale Komponente enthalten [55]. Phylloidestumoren der Mamma werden gemäss der aktuellen WHO-Klassifikation als benigne, borderline und maligne eingeteilt [55]. Entsprechend der aktuellen WHO-Klassifikation [55] hängt die Zuordnung der Kategorie von Tumorbegrenzung, Stromazellularität, Stromaatypien, mitotischer Aktivität der Stromazellen, dem sog. „stromal overgrowth“ und dem Nachweis maligner heterologer Elemente ab [16, 47, 55]. Eine eindeutige Zuordnung der Kategorie ist in der Regel erst am Operationspräparat möglich, da die Tumoren sehr heterogen aufgebaut sein können. Maligne Phylloidestumoren sind als B5d einzustufen, sofern diese in der Nadelbiopsie sicher als maligne eingeordnet werden können [1, 40, 46, 55]. Die übrigen PT sind als B3 zu klassifizieren [1, 40, 46, 55]. Eine Assoziation zu präexistierenden Fibroadenomen ist bekannt, wobei der PT auch de novo entstehen kann [55]. Molekulare Tests, um die entsprechenden Alterationen (*TERT-* oder *MED12*-Promotermutationen bei De-novo-Formen) nachzuweisen, können in Einzelfällen hilfreich sein [55]. Die Stromazellen sind oft CD34-positiv und häufig mit Nachweis von β‑Catenin nukleärer Anfärbung, wobei das Ausmass der CD34 Expression mit den PT Subtypen korrelieren kann (ist reduziert in benignen PT Formen) [53]. Die Anwendung stromaler Marker kann in Stanzbiopsien hilfreich sein, um den PT von anderen stromalen Entitäten (wie desmoidähnliche Fibromatose, PASH oder Myofibroblastom) zu unterscheiden [55]. Bei Diagnose eines Phylloidestumors in der Stanz- oder Vakuumbiopsie ist eine vollständige Exzision angezeigt [55]. Mit Blick auf die Operationsplanung sei aufgrund aktueller Literatur darauf hingewiesen, dass beim benignen PT und beim Borderline-PT die Breite des Resektionsrandes offenbar nicht mit dem Lokalrezidivrisiko korreliert. Rosenberger et al. empfehlen bei Vorliegen eines negativen Resektionsrandes bei gutartigem PT keine Nachresektion mehr und vermuten, dass beim gutartigem PT möglicherweise auch ein positiver Rand keinen Einfluss auf das Rezidivrisiko hat. Zunächst raten die Autoren aber, weitere prospektive Daten abzuwarten, um diese Hypothese zu bestätigen [43]. Benigne Phylloidestumoren und Fibroadenome (vor allem die zellulären Formen) können histologisch überlappende Eigenschaften zeigen, wodurch die Unterscheidung im Einzelfall schwierig sein kann und die definitive Diagnose in diesen Fällen erst im Operationspräparat möglich ist [40]. Diese Läsionen sind ebenfalls der B3-Kategorie zuzuordnen und sollten zum Ausschluss eines benignen PT exzidiert werden, insbesondere bei einer Grösse von > 3 cm.

## Seltene weitere B3-Läsionen

Weitere seltene histologische Entitäten, welche als B3 klassifiziert werden, sind u. a. die mukozeleähnliche Läsion (Abb. 7), die atypische apokrine Adenose und ein breites Spektrum von stromalen oder myofibroblastären Proliferaten (wie die desmoidähnliche Fibromatose, noduläre Fasziitis), deren Differenzialdiagnose unter Umständen auch eine Herausforderung darstellen kann [1, 40, 55]. Bei mukozeleähnlichen Läsionen ist es relevant, atypische epitheliale Zellen in den Schleimseen resp. ein muzinöses Karzinom immunhistochemisch auszuschliessen [55]. Bei den blande aussehenden spindelzelligen, stromalen Proliferaten stellt die desmoidähnliche Fibromatose eine wichtige Differenzialdiagnose zu einem low-grade fibromatoseähnlichen, metaplastischen Mammakarzinom und einem Phylloidestumor dar, bei dem nur die stromale Komponente erfasst wurde. Diese Abgrenzung kann mithilfe eines immunhistochemischen Panels (β-Catenin, Desmin, sm-Aktin, CD34, Panzytokeratin, p63, basale Zytokeratine) erreicht werden [55]. Aus heutiger Sicht wird bei der asymptomatischen Patientin ein primär abwartende, nichtoperative Therapiestrategie favorisiert und es wird eine spontane Regression der Fibromatose in einem Drittel der Fälle berichtet [7, 8]. Eine atypische apokrine Adenose oder Hyperplasie (B3) liegt im Spektrum apokriner Läsionen zwischen einem low-grade apokrinen DCIS (B5a-Kategorie) und einer apokrinen Adenose/Metaplasie (B2-Kategorie). Die endgültige diagnostische Abgrenzung zwischen einer atypischen apokrinen Adenose und einem apokrinen DCIS („non high-grade“) ist in den meisten Fällen erst am Operationspräparat möglich, da die Immunhistochemie (ER, CK5/6, CK14, AR) in diesem apokrinen Spektrum meist gleicht ausfällt und nicht weiter hilfreich ist [55]. Ausnahme bildet der Nachweis einer starken HER2-Überexpression (Score 3+), der für ein apokrines high-grade DCIS spricht.

**Figure 7:**